# Temperature variability in the day–night cycle is associated with further intracranial pressure during therapeutic hypothermia

**DOI:** 10.1186/s12967-017-1272-y

**Published:** 2017-08-03

**Authors:** Adriano Barreto Nogueira, Eva Annen, Oliver Boss, Faraneh Farokhzad, Christopher Sikorski, Emanuela Keller

**Affiliations:** 1 Division of Neurosurgery Clinic, Hospital das Clínicas, Faculty of Medicine, University of Sao Paulo, 255 Dr. Eneas de Carvalho Aguiar Ave, Sao Paulo, 05403-900 Brazil; 2 Neurocritical Care Unit, Department of Neurosurgery, University Hospital, University of Zurich, Frauenklinikstrasse 10, 8091 Zurich, Switzerland

**Keywords:** Intracranial pressure, Hypothermia, Circadian rhythm, Temperature, Prediction, Multimodality monitoring

## Abstract

**Background:**

To assess whether circadian patterns of temperature correlate with further values of intracranial pressure (ICP) in severe brain injury treated with hypothermia.

**Methods:**

We retrospectively analyzed temperature values in subarachnoid hemorrhage patients treated with hypothermia by endovascular cooling. The circadian patterns of temperature were correlated with the mean ICP across the following day (ICP_24_).

**Results:**

We analyzed data from 17 days of monitoring of three subarachnoid hemorrhage patients that underwent aneurysm coiling, sedation and hypothermia due to refractory intracranial hypertension and/or cerebral vasospasm. ICP_24_ ranged from 11.5 ± 3.1 to 24.2 ± 6.2 mmHg. The ratio between the coefficient of variation of temperature during the nocturnal period (18:00–6:00) and the preceding diurnal period (6:00–18:00) [temperature variability (TV)] ranged from 0.274 to 1.97. Regression analysis showed that TV correlated with ICP_24_ (Pearson correlation = −0.861, adjusted R square = 0.725, p < 0.001), and that ICP_24_ = 6 (4–TV) mmHg or, for 80% prediction interval, ${\text{ICP}}_{24} = 23.9 - 6.22\,\times\,{\text{TV }} \pm 1.73\,\times\sqrt {1.06 + (({\text{TV}} - 1.1)^{2} /4.49)}$ mmHg. The results indicate that the occurrence of ICP_24_ higher than 20 mmHg is unlikely after a day with TV ≥1.0.

**Conclusions:**

TV correlates with further ICP during hypothermia regardless the strict range that temperature is maintained. Further studies with larger series could clarify whether intracranial hypertension in severe brain injury can be predicted by analysis of oscillation patterns of autonomic parameters across a period of 24 h or its harmonics.

**Electronic supplementary material:**

The online version of this article (doi:10.1186/s12967-017-1272-y) contains supplementary material, which is available to authorized users.

## Background

Severe brain injury may lead to a progressive worsening of neurological status and consequently to poor outcome. Nonetheless, the intensity of neurological deterioration varies widely in each case and frequently is unpredictable. Such poor outcome occurs in part because an effective neuroprotective therapy is missing and the currently available therapies may lead to significant side effects. Therefore, the treatment of patients with severe brain injury could be improved by the development of new methods to predict neurological status worsening [1–3], which could guide bedside decisions in a tailored manner.

We have previously shown that the adult human brain displays a potential novel mechanism of plasticity that involves a neurogenic system orchestrated in a broad brain area [4]. This system begins from the structures without blood–brain barrier, i.e. the circumventricular organs, located principally in the hypothalamus. Next, we showed that the pattern of hypothalamic functions such as circadian rhythms and thermoregulation anticipates signs of brain injury such as seizures in epileptic patients [5], perhaps reflecting the functioning of the potential neurogenic system. Following the same rationale, here we investigated whether the temperature variability (TV) in the day–night cycle anticipates intracranial pressure (ICP) values in subarachnoid hemorrhage patients submitted to intravascular catheter-induced hypothermia. The development of this method may reveal a parameter to anticipate neurological worsening in severe acute neurological conditions.

## Methods

### Clinical data

We performed retrospective analysis from subarachnoid hemorrhage patients enrolled in the ongoing project named “ICU Cockpit”, which has been approved from local institutional review boards. Regarding the patients analyzed in this study, ICP and brain temperature (Neurovent-P, Raumedic AG, Helmbrechts, Germany) were used for brain monitoring. Body temperature was monitored by intra-arterial thermistors (PiCCO system, Pulsion Medical Systems SE, Munich, Germany). Hypothermia (target core body temperature 33.0–33.9 °C) was induced and maintained using endovascular cooling (Quattro™, Zoll Medical, Chelmsford, USA) according to a standardized protocol if intracranial hypertension and/or delayed cerebral ischemia refractory to conventional treatment occurred [1]. Glasgow outcome scale (GOS) was assessed after 1 year.

### Data analysis

Neuromonitoring and systemic data obtained with Infinity^®^ Delta Monitor (Dräger AG, Lübeck, Germany) were conveyed to and synchronized in Component Neuromonitoring System (CNS) monitor (Moberg Research, Inc., Ambler, Pennsylvania, USA). Continuous parameters were stored at 1–10 Hz. Next, files obtained from CNS monitors were converted into Matlab files (Matlab R2014a, Natick, Massachusetts, USA) for statistical analysis. We selected the cases with ICP monitoring registered during more than 5 days. Because we suspected that the circadian rhythms could be a primary factor that correlates with brain injury, we built a spreadsheet in Excel (Excel 2010, Microsoft Corporation, Redmond, Washington, USA) containing the mean and standard deviation of all parameters in diurnal (6:00–18:00) and nocturnal (18:00–6:00) periods, encompassing all days of monitoring. We used this time to define diurnal and nocturnal periods based on the pattern of intrinsic circadian rhythms, which are related to endogenous oscillation of suprachiasmatic nucleus activity and observed through parameters such as serum level of melatonin and core body temperature [6]. To exclude values that corresponded to artifact, we set upper and lower limits based on clinical knowledge and visual analysis of the graphs plotted from data of each parameter. Days with more than 15% of values out of range were excluded of the analysis. Particularly regarding the assessment of the autonomic nervous system, we obtained as parameter the RR (or NN) interval in milliseconds to calculate the heart rate variability. Regarding temperature, we included for statistical analysis days with mean daily temperature <34.9 °C. This temperature limit includes from the first day of hypothermia (target temperature equals 33–33.9 °C) until the day when slow rewarming began. In this manner, this last day of monitoring comprehended initially a period during which the target temperature for hypothermia was maintained and next a period that corresponded to the beginning of rewarming. Importantly, this rewarming was slow in such a way that the mean temperature of that day was less than 34.9 °C. Statistical analysis to find a parameter that could correlate with ICP was carried out using Excel and Minitab 17 (Minitab Inc., State College, Pennsylvania, USA). Excel was used to perform descriptive statistics, ANOVA, and regression analysis. Minitab was used to obtain the graph of regression analysis including regression line and curves of confidence and prediction intervals (80%).

## Results

From the initial series containing 11 enrolled patients in the ICU Cockpit Project, we obtained data from 17 days of monitoring during hypothermia induced in three patients with subarachnoid hemorrhage that were treated with aneurysm coiling and sedation (Table 1). The continuous parameters suitable for statistical analysis were ICP, body temperature, blood pressure, and heart rate. We did not find correlation between heart rate variability or blood pressure with further ICP (Additional files 1, 2).

**Table 1:** Clinical features

Case #	Age	Gender	Aneurysm	Admission	Clinical assessment	Fisher grade	Recording in CNS monitor	GOS after 1 year
1	50	F	ACoA	d4	HH 2 VS d6	3	From d6	5
2	65	F	ACoA	d4	HH 4	4	From d9	3
3	53	F	ACoA	d0	HH 3	4	From d6	5

*ACoA* anterior communicating artery, *CNS* Component Neuromonitoring System, *d* day after bleeding, *F* female, *GOS* Glasgow outcome scale, *HH* Hunt and Hess scale, *VS* clinical vasospasm

### Temperature variability correlated with mean ICP in the following day

**Fig. 1:**
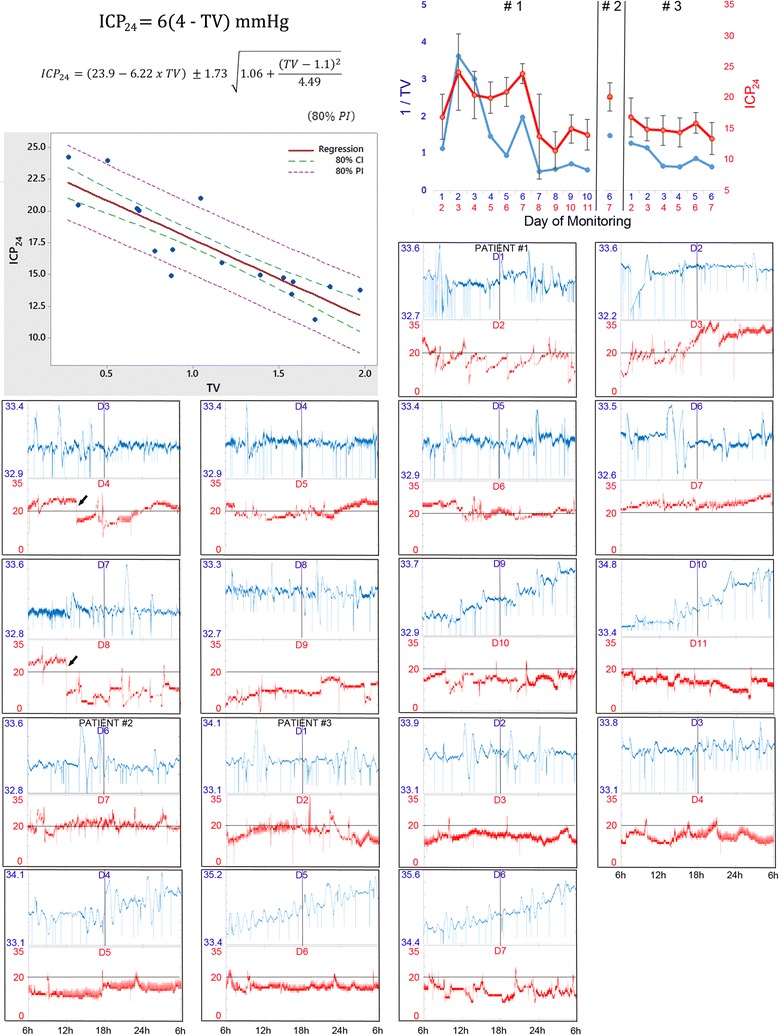
Temperature variability (TV) correlates inversely with mean intracranial pressure in the following day (ICP_24_) during hypothermia. *Upper left*-*hand graph* displays the distribution of the ICP_24_ in function of the TV from which derived the regression line and the formulas displayed above the graph. *Upper right*-*hand* graph displays daily TV and ICP_24_ of patients 1–3. Note that patient #2 underwent hypothermia during day 6 of monitoring. Broadly, the inverse of TV parallels ICP_24_. The *remaining boxes* display the daily temperature curves in degrees Celsius and the corresponding ICP curves in the following day. Temperature was recorded at 10 Hz and ICP at 1 Hz. *Vertical line* in the temperature curves corresponds to 18 h and divide the graph in day at left and night at right. *Horizontal line* in the ICP curves sets 20 mmHg. These graphs allow a qualitative analysis regarding temperature variability during day and night and ICP in the next 24 h. For example, compare the variability between day and night of days 6 and 8 of monitoring of patient #1 and the respective ICP values in the next day. *CI* confidence interval, *D* day of monitoring, *PI* prediction interval, *arrows* sudden ICP decrease due to cerebrospinal fluid drainage

The ratio between the coefficient of variation (standard deviation/mean) of temperature during the nocturnal period (18:00–6:00) and the preceding diurnal period (6:00–18:00) (TV) ranged from 0.274 to 1.97 (Table 2). Mean ICP across the following day (ICP_24_) ranged from 11.5 ± 3.1 to 24.2 ± 6.2 mmHg (Table 2). The principal result was that TV correlated inversely with ICP_24_ (Pearson correlation = −0.861, adjusted coefficient of determination R^2^ = 0.725, p < 0.001) (Fig. 1). This correlation can be visualized, for example, in the upper right-hand graph of Fig. 1, which shows that the inverse of TV parallels ICP_24_ (i.e., the mean ICP that occurs in the following 24 h).

**Table 2:** Circadian patterns of temperature and intracranial pressure under hypothermia

Case #	Day of T monitoring	Diurnal T	Nocturnal T	TV	Predicted ICP_24_	ICP_24_
1	1	33.1365 ± 0.1016	33.1805 ± 0.0897	0.882	18.5 (16.7–20.3)	16.9 ± 3.6
1	2	33.0117 ± 0.212	33.1603 ± 0.0583	0.274	22.2 (20.3–24.1)	24.2 ± 6.2
1	3	33.1089 ± 0.0634	33.1012 ± 0.021	0.331	21.9 (20–23.8)	20.5 ± 3.8
1	4	33.111 ± 0.0361	33.1035 ± 0.0247	0.684	19.7 (17.9–21.5)	20 ± 2.5
1	5	33.1142 ± 0.0388	33.1234 ± 0.0405	1.044	17.5 (15.7–19.3)	20 ± 2.3
1	6	33.1145 ± 0.1375	33.0374 ± 0.0694	0.506	20.8 (19–22.6)	24 ± 1.6
1	7	33.0858 ± 0.0476	33.0935 ± 0.0938	1.970	11.7 (9.8–13.6)	13.7 ± 6.9
1	8	33.0643 ± 0.0342	33.0427 ± 0.0584	1.709	13.3 (11.5–15.2)	11.5 ± 3.1
1	9	33.1612 ± 0.0859	33.4189 ± 0.1192	1.377	15.4 (13.6–17.2)	15 ± 2.3
1	10	33.7116 ± 0.1166	34.2782 ± 0.2091	1.764	12.9 (11–14.7)	14 ± 2.5
2	6	33.1231 ± 0.0954	33.0969 ± 0.0641	0.672	19.8 (18–21.6)	20.8 ± 2.3
3	1	33.5335 ± 0.1086	33.5147 ± 0.0845	0.779	19.1 (17.3–20.9)	16.8 ± 3.1
3	2	33.5118 ± 0.078	33.5112 ± 0.068	0.872	18.5 (16.8–20.3)	14.9 ± 1.7
3	3	33.485 ± 0.0364	33.5293 ± 0.0555	1.523	14.5 (12.7–16.3)	14.7 ± 2.4
3	4	33.4787 ± 0.0831	33.7156 ± 0.1312	1.568	14.2 (12.4–16)	14.4 ± 2.4
3	5	34.0214 ± 0.1698	34.55 ± 0.1982	1.149	16.8 (15–18.6)	15.9 ± 1.7
3	6	34.7498 ± 0.105	35.0813 ± 0.1652	1.558	14.3 (12.4–16)	13.5 ± 2.6

Artifact occurred in 0–24% (mean 2.7%) of the time regarding temperature monitoring periods of 12 h (less than 15% of the time in 24 h) and in 0–4.7% (mean 1.1%) of the time regarding ICP_24_ monitoring periods. Note that usually the predicted ICP_24_ was similar to ICP_24_, and that the predicted interval of ICP_24_ encompassed ICP_24_

*ICP*_*24*_ mean intracranial pressure in mmHg across 24 h after temperature monitoring (80% prediction interval shown between parenthesis), *T* temperature (in °C), *TV* temperature variability

### A formula correlating circadian pattern of temperature with further ICP derived from the results

From regression analysis, we obtained the formula ICP_24_ = 23.9–6.22 TV mmHg. A simplified manner to write this formula and facilitate its memorization is ICP_24_ = 6 (4–TV) mmHg. This formula determines the regression line. Complementary, to calculate the range of expected ICP_24_ with 80% of certainty for a certain day, one might use TV (for example, right after 6 a.m., when a 24 h-TV monitoring is completed) in the formula ${\text{ICP}}_{24} = 23.9 - 6.22\,\times\, {\text{TV }} \pm1.73\,\times\sqrt {1.06 + (({\text{TV}} - 1.1)^{2} /4.49)}$ mmHg. In other words, this is the formula for 80% prediction interval. For 80% confidence interval, the formula is expressed as ${\text{ICP}}_{24} = 23.9 - 6.22\,\times\,{\text{TV }} \pm 1.73\,\times\sqrt {0.06 + (({\text{TV}} - 1.1)^{2} /4.49)}$ mmHg. This confidence interval determines the slope in which the regression line fits with 80% of certainty. The graph at the upper left-hand side in Fig. 1 allows the visualization of the regression line, confidence interval (inner dashed lines), and prediction interval (outer dashed lines).

## Discussion

The analysis of the ICP curve in head injury patients has been allowed the development of prediction models with up to 30 min of antecedence [7, 8].

The present results indicate that an alteration in the circadian rhythm may anticipate intracranial hypertension. One should bear in mind that the period during which a neurological patient undergoes therapeutic hypothermia is the most critical period of treatment, when episodes of intracranial hypertension are likely to occur. The results of this study seem to be particularly useful during this period, in cases in which a strict temperature control is maintained (please note that the maximum standard deviation of temperature in 12 h periods was 0.2).

The analysis of the ICP_24_ formula may guide the therapy in severe cases of subarachnoid hemorrhage. A caveat in this analysis is that ICP_24_ is a mean ICP value, and along a 24 h period it is possible that intracranial hypertension episodes take place. However, in our results we found a variability in ICP that in general did not exceed 20% of ICP_24_. For example, the third quartile of the coefficients of variability of the 17 analyzed days equals 0.192, which means that in 75% of the analyzed days the standard deviation of ICP_24_ did not exceed 19.2% ICP_24_. In this manner, it is highly probable that the patient will experience a day with episodes of intracranial hypertension after a day with TV ≤0.333. On the other hand, a day without episodes of intracranial hypertension is highly probable after a day with TV ≥1. These predictions are suitable while the patient undergoes a relatively constant treatment and a strict mild hypothermia. Therefore, a clinical implication of this study may be that endovascular catheter-induced hypothermia may lead to more predictable ICP [9, 10].

It remains to be determined whether the circadian patterns of temperature correlate with further ICP under normothermia. A further parameter that could be tested regarding ICP prediction under normothermic conditions is the ratio between mean temperature during night and the preceding diurnal period. This parameter was not suitable for ICP prediction due to the strict temperature ranges used to treat the enrolled patients. Nonetheless, this parameter could be assessed in a further study to investigate whether in determined circumstances it may serve to predict intracranial hypertension in the same way it predicts seizure in epileptic patients with 24 h of antecedence [5].

Likewise, perhaps other autonomic parameters correlate with further ICP in a manner not revealed in this study [5]. Blood pressure and heart rate display circadian pattern similar to temperature [11]. Heart rate variability has been shown to be a prognostic factor in conditions such as myocardial infarction [12]. Maybe the balance between the activities of the parasympathetic nervous system assessed by heart rate variability and of the sympathetic nervous system assessed clinically by electrodermal activity [5] may harbor a pattern that correlates with further ICP, although we did not find a correlation between autonomic parameter other than temperature and further ICP.

A major caveat of this study is that the method proposed here remains to be improved after confirmation of the results with the analysis of larger series with patients under different conditions, inclusion of other predictive factors, determination of confounders, and technical improvement of data acquisition and analysis. Accordingly, we studied data from a total of only 17 days of monitoring of three patients. To validate our findings with a retrospective study design, it would be necessary to make up a development cohort and a validation cohort sufficiently large to yield statistically significant results [7]. Moreover, it will be interesting to analyze larger series to test prediction models with different periods, statistical analyses, and neurological parameters. An endpoint of the algorithm could be for example the time when an episode of intracranial hypertension, if any, is likely to occur [13, 14].

## Conclusions

In conclusion, the analysis of circadian patterns of autonomic functions may be a paradigm to predict intracranial hypertension in acute brain injury. Temperature seems to be the most remarkable autonomic function to this end, even when maintained in a strict range in patients under deep sedation.

## Additional files

**Additional file 1.** Data regarding heart rate, blood pressure, and ICP. This Table shows the values obtained for the variables derived from heart rate (HR) and mean blood pressure (mBP) that are the counterpart of the variables obtained for temperature. HR and mBP data from day 6 of monitoring of patient #2 and HR data from day 3 of monitoring of patient #3 displayed artifact and were not included in the Table. Legend: BPV, blood pressure variability, defined as the ratio between the standard deviation of mean blood pressure during the nocturnal period (18:00 to 6:00) and the preceding diurnal period (6:00 to 18:00); HRV, heart rate variability; HRV_d_, standard deviation of NN interval during the diurnal period; HRV_dn_, standard deviation of NN interval during 24 h; HRV_n_, standard deviation of NN interval during the nocturnal period; HRV_n/d_, ratio between standard deviation of NN interval during the nocturnal and preceding diurnal periods; ICP_24_, mean intracranial pressure during 24 h after the monitoring day of HR and BP; mBP, mean blood pressure during 24 h.

**Additional file 2.** Statistical analysis (regression analysis, ANOVA) regarding correlation between heart rate and blood pressure variables with ICP_24_. This Table shows that there is no statistical correlation between heart rate and blood pressure variables and intracranial pressure across the following day. Legend: BPV, blood pressure variability, defined as the ratio between the standard deviation of mean blood pressure during the nocturnal period (18:00 to 6:00) and the preceding diurnal period (6:00 to 18:00); HRV_dn_, standard deviation of NN interval during 24 h; HRV_n/d_, ratio between standard deviation of NN interval during the nocturnal and preceding diurnal periods; ICP_24_, mean intracranial pressure during 24 h after the monitoring day of heart rate and blood pressure.

## Abbreviations

GOS: glasgow outcome scale
ICP: intracranial pressure
ICP_24_: mean ICP across 24 h following a day of temperature monitoring
TV: temperature variability
